# Summer diarrhea in children: a monocentric French epidemiological observational study

**DOI:** 10.1038/s41598-023-42098-x

**Published:** 2023-09-12

**Authors:** Camille Mallier, Elisa Creuzet, Céline Lambert, Julien Delmas, Audrey Mirand, Emmanuelle Rochette, Stéphane Valot, Maxime Moniot, Frédéric Dalle, Cécile Henquell, Etienne Merlin, Philippe Poirier, Matthieu Verdan, Céline Nourrisson

**Affiliations:** 1 Service de Pédiatrie, CHU Clermont-Ferrand, 63000 Clermont-Ferrand, France; 2 Service de Parasitologie-Mycologie, CHU Clermont-Ferrand, 63000 Clermont-Ferrand, France; 3 DRCI, Unité de Biostatistiques, CHU Clermont-Ferrand, 63000 Clermont-Ferrand, France; 4 Service de Bactériologie, 3IHP, INSERM, CHU Clermont-Ferrand, Université Clermont Auvergne, 63000 Clermont-Ferrand, France; 5 Service de Virologie, CNR des Entérovirus et Parechovirus, 3IHP, CHU Clermont-Ferrand, 63000 Clermont-Ferrand, France; 6 LMGE UMR CNRS 6023, Equipe EPIE - Epidémiologie et Physiopathologie des Infections à Entérovirus, Faculté de Médecine, Université Clermont Auvergne, 63001 Clermont-Ferrand, France; 7 Unité CRECHE (INSERM CIC1405), Université Clermont Auvergne, 63000 Clermont-Ferrand, France; 8 Laboratoire de Parasitologie-Mycologie, Plateforme de Biologie Hospitalo-Universitaire Gérard Mack, 21000 Dijon, France; 9 Laboratoire associé du Centre National de Référence “Cryptosporidioses, microsporidies et autres protozooses digestives”, 21000 Dijon, France; 10 Laboratoire associé du Centre National de Référence “Cryptosporidioses, microsporidies et autres protozooses digestives”, 63000 Clermont-Ferrand, France; 11 AgroSup Dijon, Equipe Vin, Aliment, Microbiologie, Stress, UMR PAM L’Université de Bourgogne Franche-Comté (UBFC), 21000 Dijon, France; 12 Service de Parasitologie-Mycologie, 3IHP, INSERM, CHU Clermont-Ferrand, Université Clermont Auvergne, 63000 Clermont-Ferrand, France

**Keywords:** Infectious-disease diagnostics, Infectious diseases

## Abstract

Pediatric diarrhea is a major public health problem worldwide. In France, continuous surveillance shows a winter epidemic peak and a more modest summer recrudescence. Few studies describe the infectious agents responsible for pediatric summer diarrhea in France. The objectives were to estimate the prevalence of infectious diarrhea and describe the pathogens responsible for summer diarrhea in children; and to describe common factors that can be used as guidance on the etiology of these diarrheas. A cross-sectional, single-center, epidemiological observational study was conducted in the pediatric emergency department of a French hospital between June and September in 2019 and 2020. Multiplex gastrointestinal pathogen panels were used for diagnostics. A multiple correspondence analysis was used to determine profiles of patients. A total of 95 children were included, of whom 82.1% (78/95) were under five years old. The prevalence of infectious summer diarrhea was 81.1% (77/95, 95%CI 71.7–88.4%). A total of 126 infectious agents were detected (50.0% bacteria, 38.1% viruses, 11.9% parasites). The main enteric pathogens were enteropathogen *Escherichia coli* (24/126), rotavirus (17/126) and *Salmonella* (16/126). A co-detection was found in 51.9% (40/77) of cases. Four patient profiles, considering the severity and the pathogen involved, were highlighted.

## Introduction

With approximately 1.7 billion annual cases worldwide, diarrhea is the third cause of infant mortality in the world after perinatal conditions and lower respiratory tract infections^1^. In 2019, 370,000 diarrhea-related deaths occurred in children under five years of age, mainly in developing countries^1^. In developed countries, the use of oral rehydration solutions has greatly reduced this mortality over the past two decades.

In 30–60% of cases, the cause of the infantile acute diarrhea remains unknown, but viral and bacterial pathogens are identified in 30–70%, and 6–20% of cases, respectively, depending on the country^2–4^. In temperate countries, viral gastroenteritis predominate in winter, unlike bacterial gastroenteritis, which peak during the summer months^5^. Unfortunately, data are lacking concerning the place of parasites in infectious diarrhea in children living in developed countries.

Moreover, data are difficult to interpret and extrapolate. (i) The rare studies are essentially carried out in developing countries where the risk of contamination is high due to precarious hygiene conditions and lack of water sanitation^6,7^. These data cannot be extrapolated to developed countries where pathogen circulation and child exposition are not comparable. (ii) In general practice, by argument of frequency, the hypothesis of a viral infection is often considered without microbiological evidence^8^. Indeed, few etiologies have been associated with specific clinical, biological or anamnestic features^9^. (iii) Finally, the increasing number of traveling children and the growing population of children issue from immigration have a probable impact on the microbiological ecology of diarrhea in developed countries of these last years^10^.

In France, the epidemiological profile of acute diarrhea includes a national winter epidemic, and, in a lesser extent, a summer peak^11^. The age group most affected is that of 1–4 years^12–14^. Despite the annual surveillance of acute diarrhea in France, descriptive studies on infectious etiologies focus on winter epidemics, often in a small age group, or target a single pathogen^2,5,15^. Data on circulating pathogens during the summer season are missing. The summer period is nevertheless conducive to contamination by enteric pathogens through exposure to bathing water, consumption of raw food, travels abroad, etc.

At the same time, the development of high-performance diagnostic tools based on molecular biology has revolutionized the diagnosis of infectious diarrhea. They could theoretically replace, in part or totally, bacterial culture or microscopy and antigenic tests for the search for parasites and viruses^16^. The growing use of these new tools, in particular detecting a panel of several enteropathogens, suggests that the prevalence of certain pathogens had been underestimated until now^5,16,17^.

The main objectives of this study were (i) to estimate the prevalence of infectious diarrhea in the summer in a pediatric population admitted to the emergency department of a French hospital, and (ii) to identify the pathogens involved. The secondary objective was to describe factors (clinical, biological, or anamnestic criteria) that could direct towards the infectious agent associated with the diarrhea.

## Methods

### Study population

This cross-sectional, single-center, observational and epidemiological study was conducted in primary care center during routine care and was based on microbiological analysis of biological material collected in a non-invasive way. Children from 0 to 16 years old, presenting with diarrhea (defined as a change in the number and consistency of stools) at the pediatric emergency department of the university hospital of Clermont-Ferrand (France) between June 1 to September 30 of 2019 and 2020 were proposed to participate in the study. Children were enrolled after the signature of an informed consent by the parents or the guardians. Children without stool emission during consultation were excluded. This clinical study was approved by the research ethics committees of the Clermont-Ferrand hospital (“Comité de Protection des Personnes Sud-Est VI”, France) with the reference number AU1574. All methods were performed in accordance with the relevant guidelines and regulations. Informed consent was obtained from all participants and/or their legal guardians.

### Anamnestic and clinical data

The parents were asked to complete an anamnestic information sheet to indicate lifestyle, dietary habits, recent history of digestive disorders or similar cases in the acquaintances, patronage of collectivity, contact with animals, exposition to bathing water and recent travel. The physician had to complete a clinical information sheet specifying vital constants, number of stools per day, presence of vomiting, abdominal pains and dehydration, cares, and evolution of the patient.

### Laboratory investigations

Stool samples were collected during consultation in a sterile jar at the time of stool output or collected directly from the diaper for younger children. They were sent at room temperature to the laboratory for molecular diagnosis of virus, bacteria, and parasites infection. Briefly, 200 mg of feces were added to a tube containing 800 µL of NUCLISENS^®^ easyMAG^®^ Lysis Buffer (bioMérieux) and 0.5 mm glass beads and were subjected to grinding on the TissueLyser^®^ II (Qiagen) for mechanical lysis (30 Hz for three minutes). Samples were centrifuged (14,000 g for ten minutes) and nucleic acids from 200 µL of supernatant were extracted on the ELITe InGenius^®^ (ELITechGroup) apparatus. Nucleic acids extracts were stored at − 80 °C until use and multiple freeze–thaw cycles were avoided. Different multiplex RT-qPCR and qPCR panels were performed on the ELITe InGenius^®^ to detect bacteria: *Salmonella* sp., *Shigella* sp., *Campylobacter* sp., *Yersinia enterolitica* (RIDA^®^GENE Bacterial Stool Panel, R-Biopharm) and different pathovars of *Escherichia coli* (enteropathogenic *E. coli* (EPEC), enterohemorrhagic *E. coli* (EHEC), shigatoxin-producing *E. coli* (STEC), entero-invasive *E. coli* (EIEC)/*Shigella* sp.) (RIDA^®^GENE EHEC/EPEC, R-Biopharm); viruses: adenovirus, astrovirus, rotavirus (Gastrointestinal Viral ELITe Panel, ELITechGroup), and norovirus (Norovirus ELITe Panel, ELITechGroup); or parasites: *Giardia duodenalis*, *Cryptosporidium* sp., *Entamoeba histolytica* (RIDA^®^GENE Parasitic Stool Panel II, R-Biopharm). To complete these panels, in-house methods were also performed as previously described, targeting the microsporidia *Enterocytozoon bieneusi* and *Encephalitozoon intestinalis/hellem*^18^, and enterovirus^19^.

In case of positive result for enterovirus RT-qPCR, genotyping was performed^19^. Similarly, positive *Cryptosporidium* PCR were completed with genotyping of GP60^18^.

Microscopic examination of fecal smear was also performed for parasites search.

### Data management and statistical analysis

This cross-sectional study, conducted on a sample, should make it possible to generalize the results to the entire target population. A margin of error on the estimate is defined to calculate the number of patients required, in addition to the expected proportion of the primary outcome. Thus, for an expected proportion of infectious diarrhea of about 80%^2–5^, the inclusion of at least 97 patients would make it possible to obtain a precision of this proportion of ± 8%.

Statistical analysis were performed with Stata (version 15; StataCorp, College Station, Texas, USA) and R 3.5.1 (http://cran.r-project.org/, accessed on February 16, 2021) software. All tests were two-sided, with an alpha level set at 5%. Categorical data were expressed as number of subjects and associated percentages, and continuous data as median [25th; 75th percentiles]. The prevalence of infectious diarrhea was presented with a 95% confidence interval (95%CI). Factors associated with infectious diarrhea (and with the number of pathogens) were studied with the chi-squared test or Fisher’s exact test for categorical data, and the Mann–Whitney test for continuous data. A multiple correspondence analysis (MCA) followed by a mixed unsupervised classification (k-means clustering applied to the partition obtained from an ascending hierarchical classification using Ward’s distance) were also implemented to (i) study the relations between the modalities of the variables and (ii) determine profiles of patients (clusters of patients sharing closely similar characteristics). For this analysis, the variables were chosen according to univariate results, clinical relevance, and statistical distribution (characteristics always present or always absent were not considered). Only patients without missing data were included in the MCA, and the sample of excluded patients was compared to the sample of included patients. The four groups obtained were then compared with chi-squared tests.

## Results

### Population

**Figure 1:**
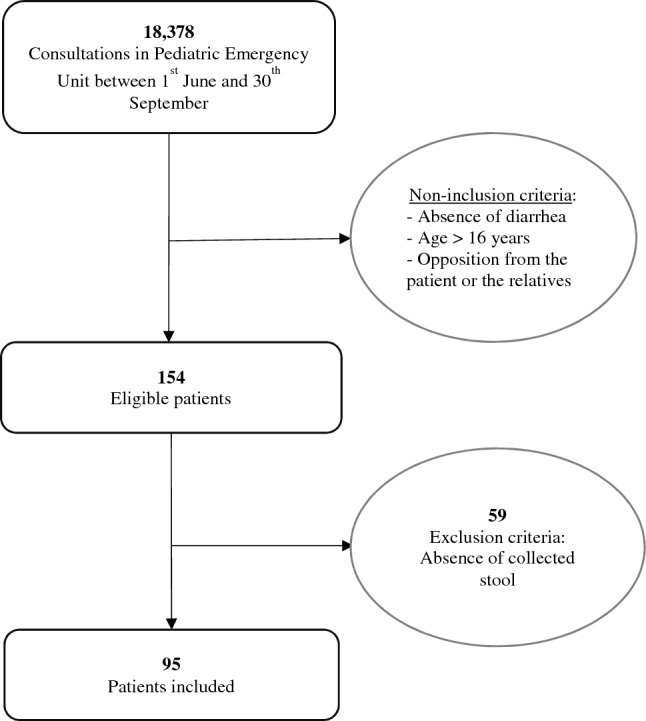
Flowchart of the study.

During inclusion period, 18,378 children were admitted to the pediatric emergency department (Fig. 1). One hundred and fifty-four (0.8%) patients were eligible but 59 did not have stool during consultation, thus 95 patients were finally included (75.8% in 2019 and 24.2% in 2020). Due to the low recruitment in 2020 and the lack of major differences observed between 2019 and 2020, the following data is provided for the two years combined. In total, 26.3% of the cohort was recruited in both June and July, the highest inclusion rate was in August (31.6%) and was then divided by two in September.

Main socio-demographic characteristics, medical data and risk factors for contamination by enteric pathogens are presented in Table S1. The median age was 22 months [11, 50], 82.1% (78/95) of patients were under five years old and 57.9% (55/95) were male. The majority of children (97.8%) lived in the region where the pediatric emergency department is located (within 50 miles).

About a half of children patronized a community including mainly a childminder and a nursery. A contact with someone with digestive disorders was reported in 25.9% (22/85) of cases, mostly in the siblings. A contact with animals was described in 65.9% (54/82) of cases, particularly with dogs or cats. A bathing water exposition concerned 62.2% (51/82) of children, mostly in private or municipal swimming pool. A travel in the previous two months was reported in 40.0% (34/85) of cases, mainly into France. Consumption of unusual foods in the two months preceding diarrhea occurred in 12.3% (10/81) of cases, including 60.0% (6/10) of artisanal dairy products.

The clinical and therapeutic characteristics of the population are presented in Table S1. The median temperature was 37.3 °C [36.9; 37.9] with a maximum at 41.1 °C. Abdominal pain was present in 53.9% (48/89) of children and clinical signs of dehydration in 41.1% (39/95). Thirty-four percent (32/94) had already received treatment before coming to the emergency department (31.3% antibiotics and 75.0% symptomatic treatment). Concerning the ten children who had received antibiotics before the consultation, the treatment prescribed was: azithromycin (n = 3), amoxicillin (n = 3), cotrimoxazole (n = 1, the children also had an abscess of the buttock), amoxicillin + clavulanic acid + cefixime (n = 1), and in two cases the parents had forgotten the name of the antibiotic.

### Laboratory investigations

**Figure 2:**
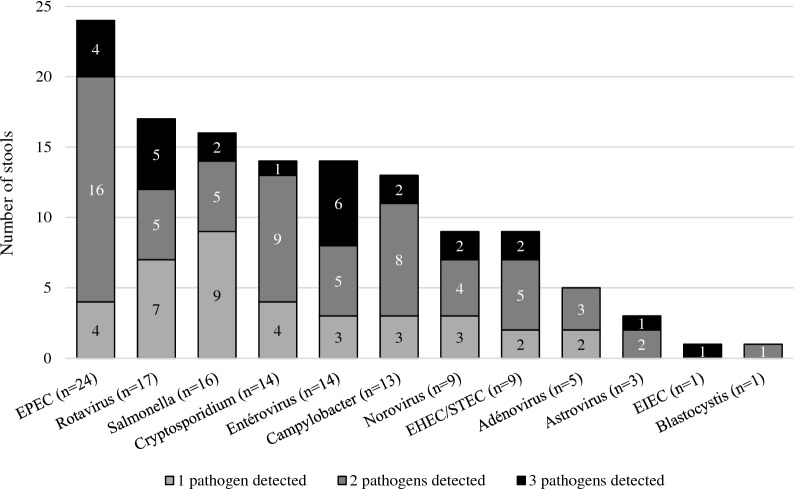
Prevalence of each enteropathogen and co-detection. The most frequently detected infectious agents were EPEC followed by rotavirus and then *Salmonella*. No *Yersinia enterocolitica*, *Giardia duodenalis*, *Entamoeba histolytica*, *Enterocytozoon bieneusi* nor *Encephalitozoon* sp. were detected. A co-detection was identified in more than half of the infectious stools. *EHEC* enterohemorrhagic *Escherichia coli*, *EIEC* enteroinvasive *Escherichia coli*, *EPEC* enteropathogen *Escherichia coli*, *STEC* Shiga toxin-producing *Escherichia coli.*

**Figure 3:**
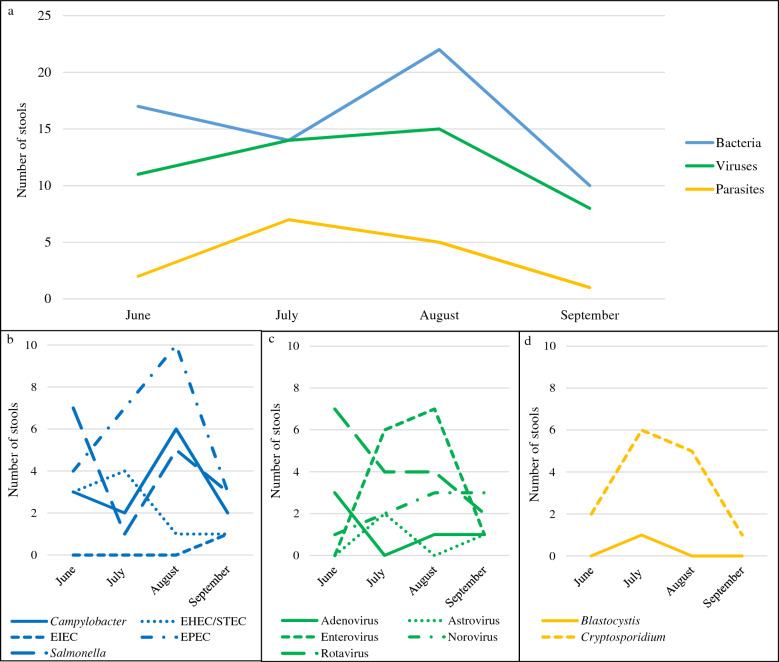
Monthly distribution of the different infectious agents detected (**a**): bacteria (**b**), viruses (**c**) and parasites (**d**). *EHEC* enterohemorrhagic *Escherichia coli*, *EIEC* enteroinvasive *Escherichia coli*, *EPEC* enteropathogen *Escherichia coli*, *STEC* Shiga toxin-producing *Escherichia coli.*

The positivity rate for a pathogen (bacteria and/or virus and/or parasite) was 81.1% (77/95, 95%CI: 71.7 to 88.4%). One hundred and twenty-six pathogens were detected in the 77 positive stools, of which 50.0% (63/126) of bacteria, 38.1% (48/126) of viruses and 11.9% (15/126) of parasites. The most common pathogens were EPEC (24/77, 31.2%), rotavirus (17/77, 22.1%) and *Salmonella* (16/77, 20.8%). A co-detection was found in 51.9% (40/77) of cases: 77.5% (31/40) with two agents and 22.5% (9/40) with three (Figs. 2 and S1). No combination of pathogens was more common than another (Table S2) and symptoms differed only on the presence of diffuse abdominal pain depending on whether one or more pathogens were detected (Table S3). Bacterial diarrhea predominated all along the study (Figs. 3a and S2a). The most frequent pathogens detected were represented in Figs. 3b–d and S2b–d. The 14 enterovirus infections were associated with different types: Enterovirus A71 (n = 4), Coxsackievirus A4, A9 and B3 (n = 2, each) and Coxsackievirus A16, B1, B2 and B4 (n = 1, each). Among the 14 *Cryptosporidium* detected, there were 13 *Cryptosporidium parvum* (7 IIaA15G2R1, 2 IIaA17G2R1, 1 IIdA17G1, 1 IIdA18G1 and 2 subtypes not identified) and 1 *Cryptosporidium hominis* (1eA11G3T3 subtype). No patient was positive for *Yersinia enterocolitica*, *Giardia intestinalis*, *Entamoeba histolytica* or microsporidia.

### Common factors and patient profiles

No anamnestic, clinical or biological factor were significantly associated with the presence of a specific infectious agent associated with diarrhea (data not shown). To complete these analyzes, a MCA was performed. Among the 95 patients, 77 were included in the MCA and 18 were excluded because of data missing from any of the variables selected for the analysis (these variables are all presented in Table 1). The 18 patients excluded were comparable to the 77 included concerning the criteria relating to age, hospitalization, dehydration or month of inclusion (data not shown). The MCA revealed four groups of patients whose characteristics are presented in Table 1. The first group (n = 33) included all patients under six months of age and the majority of patients under two. This group was characterized by an occurrence in August (no year effect). Clinically, the children of this group had few stools per day (less than five) which were more frequently mucous and were more frequently infected by a virus (rotavirus and enterovirus). Abdominal pain, weight loss and dehydration were no frequent. In the second group (n = 7), the majority of patients were between six months and two years old. Diarrhea appeared mainly in September. These children have had an infectious contact, have patronized a community setting, have bathed in recreational waters and have traveled abroad for half of them. The number of stools per day was between 5 and 10. This group included three of the seven norovirus infections. There were also some rotavirus, enterovirus, *Campylobacter* sp., EPEC and EHEC infections but no *Salmonella* sp. nor *Cryptosporidium* sp. one. The third group (n = 12) was composed of children aged two to five years old whose symptomology started mainly in July. They had less than five stools per day, without mucus nor blood and 75% of them had been in contact with animals. They were dehydrated, had weight loss and were hospitalized for more than 24 h. *Cryptosporidium* sp. diarrhea predominated in this group. Some norovirus and rotavirus infections were found, only one EHEC but no enterovirus. Finally, the fourth group (n = 25) included almost all children older than five years old. They had more than 10 bowel movements per day with mucus, vomiting, and abdominal pain that was mainly periumbilical. They had lost weight and were hospitalized for less than 24 h. Bathing and animal contact were present in this group. Most of the infections were caused by *Salmonella* sp., *Cryptosporidium* sp. and EPEC. There were a few infections with norovirus, rotavirus, *Campylobacter* sp. and EHEC and none with enterovirus.

**Table 1:** Description of the four groups obtained after the multiple correspondence analysis.

	Total (n = 77)	Group 1 (n = 33)	Group 2 (n = 7)	Group 3 (n = 12)	Group 4 (n = 25)	*p*-value
Demographic characteristics						
Month						< 0.001
June	20 (26.0)	10 (30.3)	0 (0.0)	1 (8.3)	9 (36.0)	
July	21 (27.3)	7 (21.2)	0 (0.0)	10 (83.3)	4 (16.0)	
August	25 (32.4)	14 (42.4)	1 (14.3)	1 (8.3)	9 (36.0)	
September	11 (14.3)	2 (6.1)	6 (85.7)	0 (0.0)	3 (12.0)	
Age						< 0.001
< 6 months	8 (10.4)	8 (24.2)	0 (0.0)	0 (0.0)	0 (0.0)	
6 months to 2 years	33 (42.8)	22 (66.7)	4 (57.1)	1 (8.3)	6 (24.0)	
2–5 years	22 (28.6)	3 (9.1)	2 (28.6)	10 (83.3)	7 (28.0)	
≥ 5 years	14 (18.2)	0 (0.0)	1 (14.3)	1 (8.3)	12 (48.0)	
Male sex	45 (58.4)	20 (60.6)	1 (14.3)	9 (75.0)	15 (60.0)	0.08
Risk factors						
Patronage of collectivity	38 (49.3)	17 (51.5)	6 (85.7)	3 (25.0)	12 (48.0)	0.09
Infectious contact	18 (23.4)	7 (21.2)	6 (85.7)	1 (8.3)	4 (16.0)	0.002
Contact with animals	51 (66.2)	18 (54.5)	3 (42.9)	9 (75.0)	21 (84.0)	0.048
Bathing	50 (64.9)	14 (42.4)	7 (100)	9 (75.0)	20 (80.0)	0.002
Recent travel (< 2 months) abroad	12 (15.6)	3 (9.1)	4 (57.1)	2 (16.7)	3 (12.0)	0.03
Symptoms reported or present at admission						
Number of stools per day						0.001
< 5	32 (41.5)	19 (57.6)	2 (28.6)	7 (58.3)	4 (16.0)	
Between 5 and 10	26 (33.8)	9 (27.3)	5 (71.4)	4 (33.3)	8 (32.0)	
> 10	19 (24.7)	5 (15.1)	0 (0.0)	1 (8.3)	13 (52.0)	
Mucus diarrhea	24 (31.2)	10 (30.3)	1 (14.3)	0 (0.0)	13 (52.0)	0.006
Bloody diarrhea	12 (15.6)	4 (12.1)	1 (14.3)	0 (0.0)	7 (28.0)	0.14
Vomiting	51 (66.2)	15 (45.5)	5 (71.4)	11 (91.7)	20 (80.0)	0.006
Abdominal pain	41 (53.2)	6 (18.2)	4 (57.1)	8 (66.7)	23 (92.0)	< 0.001
Periumbilical	24 (31.2)	1 (3.0)	3 (42.9)	4 (33.3)	16 (64.0)	< 0.001
Diffuse	8 (10.4)	4 (12.1)	0 (0.0)	3 (25.0)	1 (4.0)	0.21
Other localization	11 (14.3)	0 (0.0)	0 (0.0)	1 (8.3)	10 (40.0)	< 0.001
Dehydration	32 (41.6)	9 (27.3)	2 (28.6)	10 (83.3)	11 (44.0)	0.007
Recent weight loss	47 (61.0)	13 (39.4)	5 (71.4)	10 (83.3)	19 (76.0)	0.009
Medical care at emergency department						
Hospitalization	47 (61.0)	16 (48.5)	3 (42.9)	12 (100)	16 (64.0)	0.005
> 24 h	16 (20.8)	6 (18.2)	0 (0.0)	9 (75.0)	1 (4.0)	< 0.001
Infectious agents						
Norovirus	7 (9.1)	1 (3.0)	3 (42.9)	2 (16.7)	1 (4.0)	0.008
Rotavirus	14 (18.2)	9 (27.3)	3 (42.9)	1 (8.3)	1 (4.0)	0.02
Enterovirus	11 (14.3)	10 (30.3)	1 (14.3)	0 (0.0)	0 (0.0)	0.003
*Campylobacter*	10 (13.0)	4 (12.1)	3 (42.9)	0 (0.0)	3 (12.0)	0.09
*Salmonella*	11 (14.3)	1 (3.0)	0 (0.0)	0 (0.0)	10 (40.0)	< 0.001
EPEC	19 (24.7)	6 (18.2)	3 (42.9)	0 (0.0)	10 (40.0)	0.02
EHEC	8 (10.4)	3 (9.1)	1 (14.3)	1 (8.3)	3 (12.0)	0.95
*Cryptosporidium*	12 (15.6)	1 (3.0)	0 (0.0)	4 (33.3)	7 (28.0)	0.007

Data are presented as number of patients (percentages). Based on multiple correspondence analysis, four clusters (“groups”) of patients were identified as sharing closely similar characteristics.

## Discussion

Epidemiological data on acute gastroenteritis, particularly in industrialized countries, are scanty. Here, we carried out a broad screening of several enteropathogens in a systematic way for any children presenting with diarrhea during the summer period in the pediatric emergency department of a French hospital. We detected an enteropathogen in 81% of cases (95% CI 72–88%), mostly bacteria, which is comparable to previous studies^5,20^. In more than half of positive stools we identified at least two pathogens which is also consistent with previous studies, although none data are available independently for each season^2,9,21,22^. Such frequent detection of several enteropathogens simultaneously is not surprising since contamination occurs for all by the same routes (direct or indirect fecal–oral route).

In general, apart in case of dysenteric syndrome, septic state, return from overseas, immunocompromised or debilitated patients, shigellosis among relatives, or food poisoning in a community, the cause of the diarrhea is not explored. The hypothesis of a viral infection is then often retained without microbiological proof^8^. However, an accurate identification of enteric pathogens could influence therapeutic decisions, in particular concerning the relevance of antibiotics, and so will help to improve infectious diarrhea management in health care settings^23^. Another major advantage of pathogen identification is the possibility of identifying the source of contamination and therefore potentially preventing transmission or limiting epidemic phenomena. It would therefore be interesting to be able to predict the causative pathogen in a child presenting with diarrhea. Although we did not identify specific symptoms, history or biological abnormalities associated with a specific pathogen, four patient profiles emerged in our study. Groups 1 and 2 appear to bring together younger children under two years of age presenting with the mildest symptoms and mainly virus-infected. Rotavirus were the most frequently identified viruses, which is consistent with previous study^20^. The second most common viruses were enterovirus, as expected since summer epidemics are reported^19^. Due to the low number of enterovirus infections, the genotypes identified could not be correlated with epidemiology throughout France. Nevertheless, as at the national level, the enterovirus 71 was one of the major genotypes identified on the same period^24^.

At the opposite, groups 3 and 4 gather older children with more severe symptoms and more frequent and longer hospitalizations than in the previous groups and for whom bacteria or *Cryptosporidium* sp. were more frequently detected, although a study carried out in Bulgaria reported higher morbidity associated with viruses^20^. Almost all children over five years old belong to groups 3 and 4, this can be explained by the fact that these children will only consult the emergency department for more severe symptoms than an infant. Of note, mucus and/or blood were not frequent in stool, contrary to what is usually described for bacterial diarrhea^5^. In this study, EPEC were the most common pathogenic bacteria, but were associated with at least one other enteropathogen in 83% of cases. These co-infections ask about the role of EPEC in symptoms occurrence, as Lima et *al.* found 17% of EPEC in the stools of asymptomatic children^25^. As previously described, the second most detected bacterial genus was *Salmonella* sp.^5,21^. Interestingly, it is also in these groups that the notion of animal contact is the most present, which could be consistent with a higher prevalence of cryptosporidiosis than in groups 1 and 2. It is known that 80% of cryptosporidiosis occur in summer, with a peak of cases under five years of age, and animal exposure is sometimes a risk factor^26^. We detected *Cryptosporidium* sp. in 18% of infectious diarrhea, which is higher than recent data in the literature^27^. The identified genotypes were those usually circulating in France.

Our study has several limitations. Firstly, inclusions between June and September 2020 were strongly impacted by the COVID-19 pandemic (a 15% decrease in pediatric emergency department admissions was observed in our hospital). In an unplanned way, this study shows the effect of the pandemic on the occurrence of infectious diarrhea in children. Because of the French confinement, the partial return to the community, but also the widespread introduction of barrier measures and the limitation of travel, children were spared by the etiologies responsible for diarrhea at this time. Thus, this study allows us to identify mainly indigenous diarrhea. Secondly, there is also a selection bias. We included only stools collected in the emergency department, excluding 59 children that did not have stool during their coming. Thus, children with relatively few stools per day were less likely to exonerate during the consultation, especially if they were not hospitalized. So we may have included children presenting with more severe forms, but we do not have the characteristics of the 59 children excluded and so we cannot verify their comparability with the 95 children included. Thirdly, PCR positivity is not always synonym of infection, and the high percentage of co-infections in our study questions the involvement of each infectious agent in the symptomatology, notably symptoms were not more severe in children for whom multiple pathogens were detected^16^. The significance of a weakly positive PCR is not known and can reflect asymptomatic carriage, prolonged shedding, or presence of latent virus. In order to establish these Ct thresholds, it would be necessary to perform a study with a control group free of any digestive symptoms. In a study using RT-PCR to test for enteropathogens in the stool of diarrheic and non-diarrheic children, Elfving et al. found lower Ct values in sick children than in healthy ones, which allowed them to define an interpretation threshold for each target^22^. Furthermore, a negative PCR does not mean that the diarrhea is not of infectious origin because these tools are limited to a predefined target panel. *Clostridioides difficile* and sapovirus, which were not in our test panel, were detected in previous studies in 16 and 6% of diarrheal stools of children under 18 years old, respectively^5^. Fourthly, this study is not representative of the entire pediatric population as it is monocentric and took place in a hospital, so the most severe patients are encountered. Moreover, 82% of the patients included were under five years old, probably also in connection with the symptoms of dehydration more frequent in infants than in older children. So, it would be interesting to extend the inclusion of patients to general practitioners and pediatricians. More generally, it would be interesting to include patients throughout the year to assess the effect of the seasons on the occurrence of infectious diarrhea and the pathogens responsible for it. This type of study would also help to strengthen knowledge of the factors that can impact the occurrence of infectious diarrhea and the pathogens involved. For example, breastfeeding has been shown to be protective against gastrointestinal infections in young children^28^, but it is unclear whether the effect is different depending on the pathogen involved.

To conclude, our study highlights a strong circulation and a wide diversity of enteric pathogens among children in the summer, even in a developed country. No obvious characteristic associated with a particular pathogen is observed, but we suggest a higher morbidity in bacterial and parasitic infections. The high rate of co-infections suggests the need for other studies to better interpret their short-term impact (i.e. their imputability in the episode of gastroenteritis), but also their medium and long term consequences on the intestinal microbiota^29^, and the occurrence of chronic diseases such as irritable bowel syndrome^30^, or neurocognitive development disorders^31^.

### Supplementary Information

Supplementary Figures.

Supplementary Tables.

## Data Availability

The datasets generated during and/or analysed during the current study are available from the corresponding author on reasonable request.
