# Supplementary material for: Summer diarrhea in children: a monocentric French epidemiological observational study
Source: Sci Rep. 2023 Sep 12;13:15078. doi: 10.1038/s41598-023-42098-x (PMC10497495; doi:10.1038/s41598-023-42098-x)
Supplement: Supplementary file 1 — Supplementary Figures. [file 41598_2023_42098_MOESM1_ESM.pdf]

Title: Summer diarrhea in children: a monocentric French epidemiological observational study.

Running title: Infectious summer diarrhea in children.

Authors: Camille Mallier, MD[1,$] & Elisa Creuzet[2,$], Céline Lambert, MSc[3], Julien Delmas, PharmD, PhD[4], Audrey Mirand, PharmD, PhD[5,6], Emmanuelle Rochette, PhD[1,7], Stéphane Valot, PharmD[8,9], Maxime Moniot, PharmD[2], Frédéric Dalle, PharmD, PhD[8,9,10], Cécile Henquell, PharmD, PhD[5,6], Etienne Merlin, MD, PhD[1,7], Philippe Poirier, PharmD, PhD[11], Matthieu Verdan, MD[1,§] & Céline Nourrisson, PharmD, PhD[11,§,*]

Figure S1. Prevalence of each enteropathogen and co-detection according to the year.

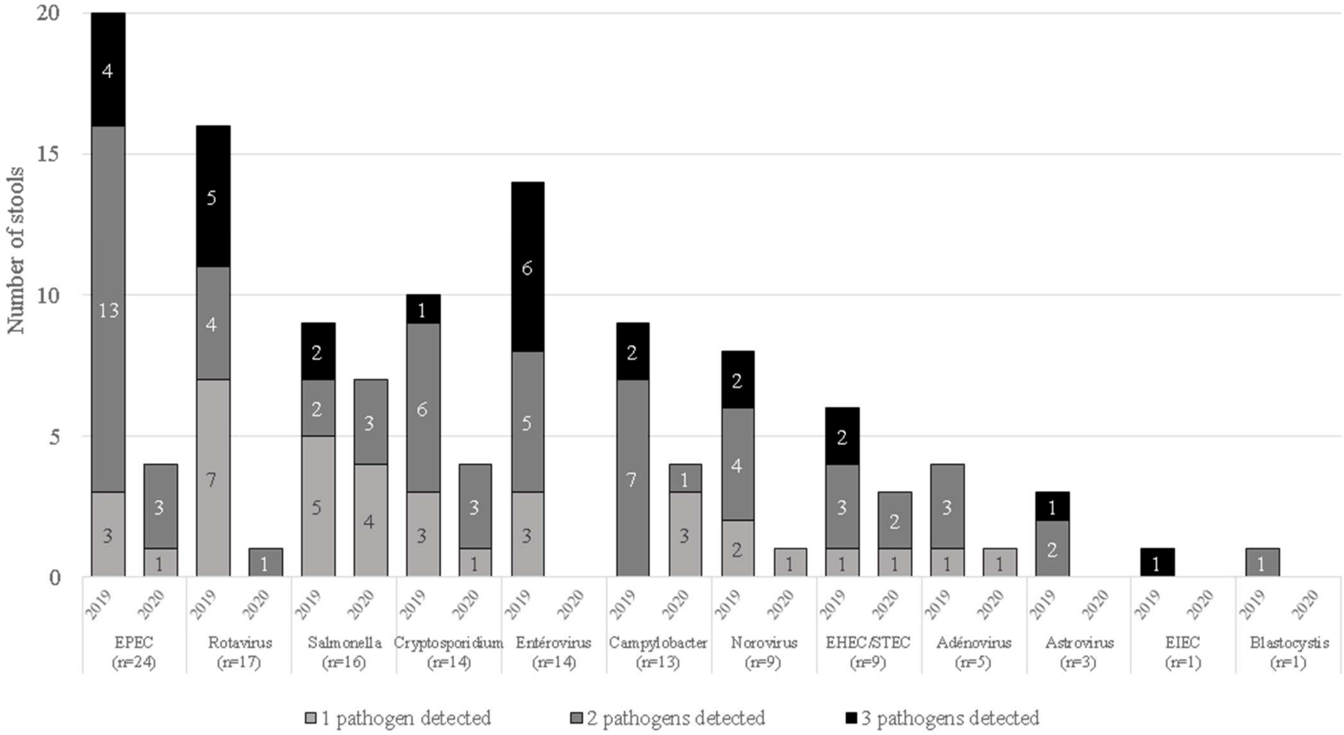

EHEC: enterohemorrhagic *Escherichia coli*; EIEC: enteroinvasive *Escherichia coli*; EPEC: enteropathogen *Escherichia coli*; STEC: Shiga toxin-producing *Escherichia coli*.

Figure S2. Monthly distribution of the different infectious agents detected according to the year (a): bacteria (b), viruses (c) and parasites (d).

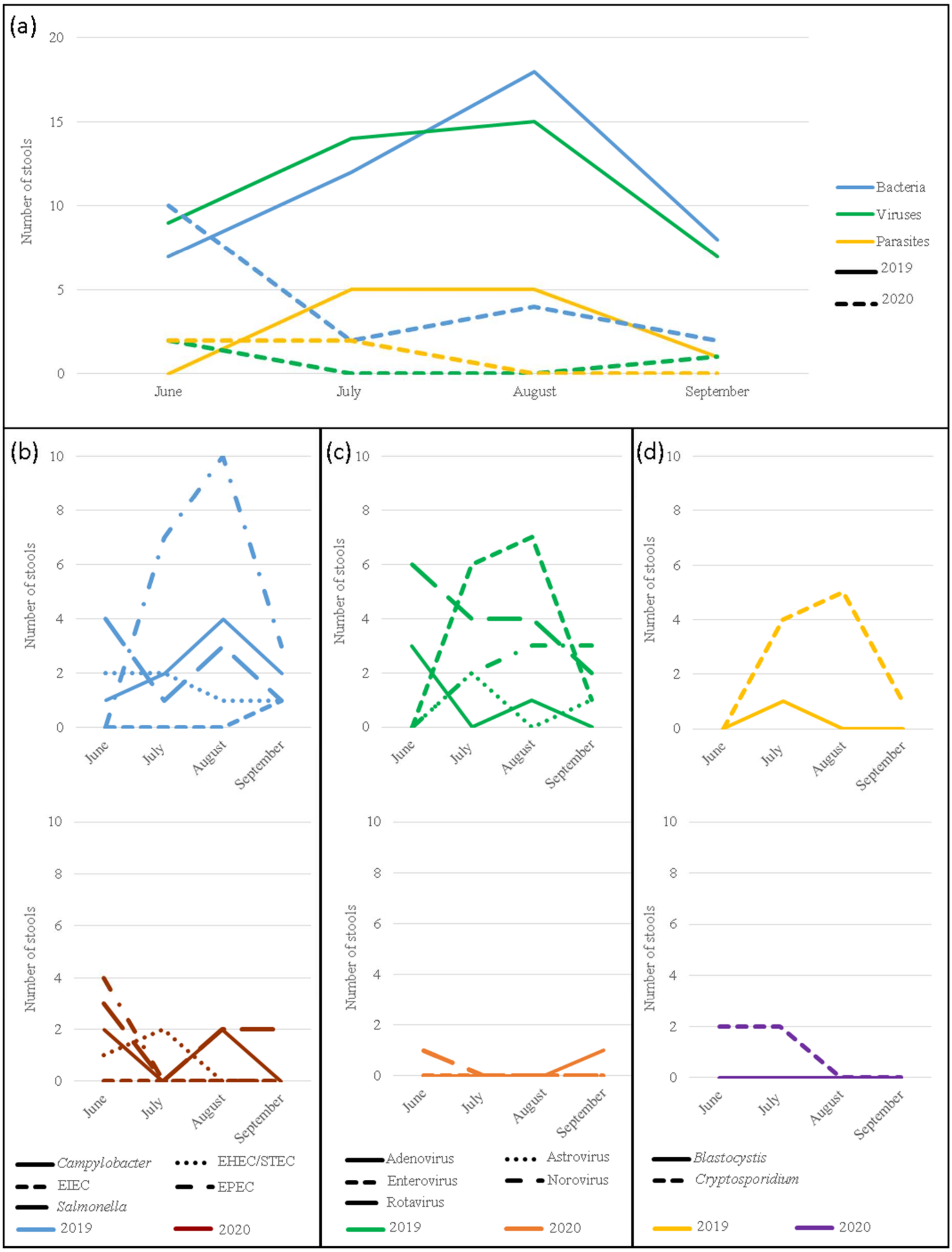

EHEC: enterohemorrhagic *Escherichia coli*; EIEC: enteroinvasive *Escherichia coli*; EPEC: enteropathogen *Escherichia coli*; STEC: Shiga toxin-producing *Escherichia coli*.
