# Supplementary material for: Summer diarrhea in children: a monocentric French epidemiological observational study
Source: Sci Rep. 2023 Sep 12;13:15078. doi: 10.1038/s41598-023-42098-x (PMC10497495; doi:10.1038/s41598-023-42098-x)
Supplement: Supplementary file 2 — Supplementary Tables. [file 41598_2023_42098_MOESM2_ESM.docx]

**Title:** Summer diarrhea in children: a monocentric French epidemiological observational study.

**Running title**: Infectious summer diarrhea in children.

**Authors:** Camille Mallier, MD^1,$^ & Elisa Creuzet^2,$^, Céline Lambert, MSc^3^, Julien Delmas, PharmD, PhD^4^, Audrey Mirand, PharmD, PhD^5,6^, Emmanuelle Rochette, PhD^1,7^, Stéphane Valot, PharmD^8,9^, Maxime Moniot, PharmD^2^, Frédéric Dalle, PharmD, PhD^8,9,10^, Cécile Henquell, PharmD, PhD^5,6^, Etienne Merlin, MD, PhD^1,7^, Philippe Poirier, PharmD, PhD^11^, Matthieu Verdan, MD^1,§^ & Céline Nourrisson, PharmD, PhD^11,§,*^

**Supplementary tables**

**Table S1.** Main characteristics of the population (n=95 patients), according to the year.

**Table S2.** Simultaneous detection of multiple pathogens (n=40) and their frequency, according to the year.

**Table S3.** Symptoms reported or present at admission, according to the number of pathogens (n=77).

**Table S1. Main characteristics of the population (n=95 patients), according to the year.** Data are presented as number of patients (percentages) or median [25th; 75th percentiles]. In the first column, “n” is the number of available data in each group (Total/2019/2020) when there is missing data. * Vaccination not compulsory in France but recommended.

|  | **Total**  **(n=95)** | **2019**  **(n=72)** | **2020**  **(n=23)** |
| --- | --- | --- | --- |
| **Demographic characteristics** |  |  |  |
| **Age** |  |  |  |
| < 6 months | 11 (11.6) | 9 (12.5) | 2 (8.7) |
| 6 months to 2 years | 41 (43.2) | 34 (47.2) | 7 (30.4) |
| 2 to 5 years | 26 (27.4) | 18 (25.0) | 8 (34.8) |
| ≥ 5 years | 17 (17.9) | 11 (15.3) | 6 (26.1) |
| **Male sex** | 55 (57.9) | 44 (61.1) | 11 (47.8) |
| **Residence department** |  |  |  |
| Region where the hospital is located (Auvergne) | 90/92 (97.8) | 70 (97.2) | 20/20 (100) |
| Department where the hospital is located (Puy-de-Dôme) | 85/92 (92.4) | 66 (91.7) | 19/20 (95.0) |
| **Medical data** |  |  |  |
| **Up to date compulsory vaccinations** | 79/89 (88.8) | 60/67 (89.6) | 19/22 (86.4) |
| **Up to date vaccination against Rotavirus*** | 4/81 (4.9) | 2/60 (3.3) | 2/21 (9.5) |
| **Immune deficiency** | 1/95 (1.1) | 1/72 (1.4) | 0/23 (0.0) |
| **Risk factors for contamination by digestive pathogens** |  |  |  |
| **Patronage of collectivity** | 39/83 (47.0) | 34/66 (51.5) | 5/17 (29.4) |
| Childminder | 14/39 (35.9) | 12/34 (35.3) | 2/5 (40.0) |
| Nursery | 10/39 (25.6) | 10/34 (29.4) | 0/5 (0.0) |
| Recreation center | 6/39 (15.4) | 6/34 (17.6) | 0/5 (0.0) |
| School | 5/39 (12.8) | 3/34 (8.8) | 2/5 (40.0) |
| Housed in a home or a medical institute | 4/39 (10.3) | 3/34 (8.8) | 1/5 (20.0) |
| **Infectious contact** | 22/85 (25.9) | 19/67 (28.4) | 3/18 (16.7) |
| Siblings | 12/22 (54.5) | 10/19 (52.6) | 2/3 (66.7) |
| Parents | 7/22 (31.8) | 6/19 (31.6) | 1/3 (33.3) |
| Close family | 6/22 (27.3) | 5/19 (26.3) | 1/3 (33.3) |
| **Contact with animals** | 54/82 (65.9) | 45/65 (69.2) | 9/17 (52.9) |
| Dogs | 38/54 (70.4) | 33/45 (73.3) | 5/9 (55.6) |
| Cats | 30/54 (55.6) | 24/45 (53.3) | 6/9 (66.7) |
| Farm animals | 14/54 (25.9) | 10/45 (22.2) | 4/9 (44.4) |
| Rabbits | 11/54 (20.4) | 9/45 (20.0) | 2/9 (22.2) |
| Reptiles | 2/54 (3.7) | 2/45 (4.4) | 0/9 (0.0) |
| Guinea pig | 1/54 (1.9) | 0/45 (0.0) | 1/9 (11.1) |
| **Consumption of unusual foods in the previous 2 months** | 10/81 (12.3) | 9/64 (14.1) | 1/17 (5.9) |
| Artisanal dairy products | 6/10 (60.0) | 6/9 (66.7) | 0/1 (0.0) |
| Dietary diversification | 2/10 (20.0) | 2/9 (22.2) | 0/1 (0.0) |
| Wild plants | 1/10 (10.0) | 1/9 (11.1) | 0/1 (0.0) |
| Spelt | 1/10 (10.0) | 1/9 (11.1) | 0/1 (0.0) |
| Spices | 1/10 (10.0) | 0/9 (0.0) | 1/1 (100) |
| **Bathing** | 51/82 (62.2) | 42/65 (64.6) | 9/17 (52.9) |
| River | 7/51 (13.7) | 6/42 (14.3) | 1/9 (11.1) |
| Lake or pond | 8/51 (15.7) | 8/42 (19.0) | 0/9 (0.0) |
| Sea or ocean | 19/51 (37.3) | 17/42 (40.5) | 2/9 (22.2) |
| Aqua park | 5/51 (9.8) | 5/42 (11.9) | 0/9 (0.0) |
| Municipal swimming pool | 19/51 (37.3) | 17/42 (40.5) | 2/9 (22.2) |
| Private swimming pool | 31/51 (60.8) | 23/42 (54.8) | 8/9 (88.9) |
| **Recent travel (< 2 months)** | 34/85 (40.0) | 32/68 (47.1) | 2/17 (11.8) |
| France | 22/34 (64.7) | 21/32 (65.6) | 1/2 (50.0) |
| Outside of France | 14/34 (41.2) | 13/32 (40.6) | 1/2 (50.0) |
| **Symptoms reported or present at admission** |  |  |  |
| **Duration of diarrhea (days)** | 3 [1; 6] | 3 [1; 6] | 3 [2; 5] |
| **Number of stools per day** |  |  |  |
| < 5 | 41 (43.2) | 32 (44.4) | 9 (39.1) |
| Between 5 and 10 | 31 (32.6) | 23 (31.9) | 8 (34.8) |
| > 10 | 23 (24.2) | 17 (23.6) | 6 (26.1) |
| **Mucus diarrhea** | 30/92 (32.6) | 23/71 (32.4) | 7/21 (33.3) |
| **Bloody diarrhea** | 16/93 (17.2) | 11 (15.3) | 5/21 (23.8) |
| **Vomiting** | 60 (63.2) | 45 (62.5) | 15 (65.2) |
| < 5 | 47/59 (79.7) | 34/44 (77.3) | 13/15 (86.7) |
| Between 5 and 10 | 7/59 (11.9) | 6/44 (13.6) | 1/15 (6.7) |
| > 10 | 5/59 (8.5) | 4/44 (9.1) | 1/15 (6.7) |
| **Body temperature in °C (n=84/63/21)** | 37.3 [36.9; 37.9] | 37.3 [36.7; 37.9] | 37.5 [37.0; 38.0] |
| **Fever (body temperature ≥ 38°C)** | 16/84 (19.0) | 11/63 (17.5) | 5/21 (23.8) |
| **Abdominal pain** | 48/89 (53.9) | 36/68 (52.9) | 12/21 (57.1) |
| Periumbilical | 27/48 (56.3) | 19/36 (52.8) | 8/12 (66.7) |
| Diffuse | 12/48 (25.0) | 10/36 (27.8) | 2/12 (16.7) |
| Other localization | 12/48 (25.0) | 8/36 (22.2) | 4/12 (33.3) |
| **Dehydration** | 39 (41.1) | 26 (36.1) | 13 (56.5) |
| **Recent weight loss** | 54/93 (58.1) | 39 (54.2) | 15/21 (71.4) |
| < 5% of body weight | 27/54 (50.0) | 19/39 (48.7) | 8/15 (53.3) |
| Between 5 and 10% of body weight | 20/54 (37.0) | 13/39 (33.3) | 7/15 (46.7) |
| > 10% of body weight | 7/54 (13.0) | 7/39 (18.0) | 0/15 (0.0) |
| **Antibiotic treatment before admission** | 10/94 (10.6) | 8 (11.1) | 2/22 (9.1) |
| **Medical care at emergency department** |  |  |  |
| **Blood sampling** | 58 (61.1) | 41 (56.9) | 17 (73.9) |
| **Hospitalization** | 57 (60.0) | 39 (54.2) | 18 (78.3) |
| Duration of stay (days) | 1 [1; 2] | 1 [1; 3] | 1 [1; 2] |
| > 24 hours | 23/57 (40.4) | 15/39 (38.5) | 8/18 (44.4) |
| Stay in intensive care unit | 2/57 (3.5) | 2/39 (5.1) | 0/18 (0.0) |
| **Intravascular filling** | 6/56 (10.7) | 5/38 (13.2) | 1/18 (5.6) |
| **Intravenous hydration** | 47/56 (83.9) | 29/38 (76.3) | 18/18 (100) |
| **Antibiotics prescription** | 7/56 (12.5) | 5/38 (13.2) | 2/18 (11.1) |

**Table S2. Simultaneous detection of multiple pathogens (n=40) and their frequency, according to the year.** Data are presented as number of stool (percentages).

| **Co-infections** | **Total**  **(n=40)** | **2019**  **(n=34)** | **2020**  **(n=6)** |
| --- | --- | --- | --- |
| **Viral** | **4 (10.0)** | **4 (11.8)** | **0 (0.0)** |
| Rotavirus + enterovirus | 1 | 1 | 0 |
| Rotavirus + enterovirus + astrovirus | 1 | 1 | 0 |
| Norovirus + astrovirus | 1 | 1 | 0 |
| Rotavirus + adenovirus | 1 | 1 | 0 |
| **Bacterial** | **8 (20.0)** | **6 (17.6)** | **2 (33.3)** |
| EHEC + *Salmonella* | 1 | 1 | 0 |
| EHEC + *Campylobacter* | 1 | 1 | 0 |
| EPEC + *Salmonella* | 2 | 1 | 1 |
| EPEC + *Campylobacter* | 4 | 3 | 1 |
| **Parasitic** | **1 (2.5)** | **1 (2.9)** | **0 (0.0)** |
| *Cryptosporidium* + *Blastocystis* | 1 | 1 | 0 |
| **Bacterial and viral** | **18 (45.0)** | **17 (50.0)** | **1 (16.7)** |
| *Salmonella* + enterovirus + norovirus | 1 | 1 | 0 |
| *Salmonella* + adenovirus | 1 | 1 | 0 |
| *Salmonella* + rotavirus | 1 | 0 | 1 |
| *Campylobacter* + adenovirus | 1 | 1 | 0 |
| *Campylobacter* + enterovirus | 2 | 2 | 0 |
| EPEC + enterovirus | 2 | 2 | 0 |
| EPEC + rotavirus | 1 | 1 | 0 |
| EPEC + norovirus | 3 | 3 | 0 |
| EPEC + enterovirus + rotavirus | 2 | 2 | 0 |
| EPEC + *Campylobacter* + norovirus | 1 | 1 | 0 |
| EPEC + *Campylobacter* + rotavirus | 1 | 1 | 0 |
| EHEC + *Salmonella* + rotavirus | 1 | 1 | 0 |
| EIEC/*Shigella* + rotavirus + enterovirus | 1 | 1 | 0 |
| **Bacterial and parasitic** | **7 (17.5)** | **4 (11.8)** | **3 (50.0)** |
| EHEC + *Cryptosporidium* | 3 | 1 | 2 |
| EPEC + *Cryptosporidium* | 4 | 3 | 1 |
| **Viral and parasitic** | **1 (2.5)** | **1 (2.9)** | **0 (0.0)** |
| Astrovirus + *Cryptosporidium* | 1 | 1 | 0 |
| **Bacterial, viral and parasitic** | **1 (2.5)** | **1 (2.9)** | **0 (0.0)** |
| EHEC + enterovirus + *Cryptosporidium* | 1 | 1 | 0 |

**Table S3. Symptoms reported or present at admission, according to the number of pathogens (n=77).** Data are presented as number of patients (percentages) or median [25th; 75th percentiles]. In the first column, “n” is the number of available data in each group (1 pathogen/≥ 2 pathogens) when there is missing data.

|  | **1 pathogen**  **(n=37)** | **≥ 2 pathogens**  **(n=40)** | ***p-value*** |
| --- | --- | --- | --- |
| **Symptoms reported or present at admission** |  |  |  |
| **Duration of diarrhea (days)** | 3 [2; 4] | 4 [2; 6] | *0.23* |
| **Number of stools per day** |  |  |  |
| < 5 | 11 (29.7) | 19 (47.5) | *0.28* |
| Between 5 and 10 | 14 (37.8) | 11 (27.5) |  |
| > 10 | 12 (32.4) | 10 (25.0) |  |
| **Mucus diarrhea** | 12 (32.4) | 15/37 (40.5) | *0.47* |
| **Bloody diarrhea** | 8 (21.6) | 8/38 (21.1) | *0.95* |
| **Vomiting** | 21 (56.8) | 27 (67.5) | *0.33* |
| < 5 | 15/21 (71.4) | 21/26 (80.8) | *0.52* |
| Between 5 and 10 | 3/21 (14.3) | 4/26 (15.4) |  |
| > 10 | 3/21 (14.3) | 1/26 (3.8) |  |
| **Body temperature in °C (n=34/34)** | 37.5 [37.0; 38.0] | 37.2 [36.6; 37.9] | *0.22* |
| **Fever (body temperature ≥ 38°C)** | 7/34 (20.6) | 5/34 (14.7) | *0.53* |
| **Abdominal pain** | 23/34 (67.6) | 21/38 (55.3) | *0.28* |
| Periumbilical | 13/23 (56.5) | 13/21 (61.9) | *0.72* |
| Diffuse | 9/23 (39.1) | 2/21 (9.5) | *0.02* |
| Other localization | 5/23 (21.7) | 6/21 (28.6) | *0.60* |
| **Dehydration** | 17 (45.9) | 15 (37.5) | *0.45* |
| **Recent weight loss** | 20/35 (57.1) | 26 (65.0) | *0.49* |
| < 5% of body weight | 12/20 (60.0) | 10/26 (38.5) | *0.35* |
| Between 5 and 10% of body weight | 5/50 (25.0) | 12/26 (46.1) |  |
| > 10% of body weight | 3/20 (15.0) | 4/26 (15.4) |  |
| **Antibiotic treatment before admission** | 4/36 (11.1) | 3 (7.5) | *0.70* |
